# Sustainable elements of the ongoing growth in the demand for halal products in OBOR regional markets

**DOI:** 10.3389/fnut.2025.1533322

**Published:** 2025-05-01

**Authors:** Hasnain Abbas, Gang Tian, Narmeen Faiz, Mengyin Jiang, Hafeez Ullah

**Affiliations:** ^1^School of Management, Institute of Management Engineering, Jiangsu University, Zhenjiang, China; ^2^Department of Management Science and Engineering, School of Economics and Management, Southeast University, Nanjing, Jiangsu, China; ^3^Sichuan University of Arts and Science, Tongchuan, Sichuan, China; ^4^Antai College of Economics and Management, Shanghai Jiao Tong University, Shanghai, China

**Keywords:** intelligent manufacturing, halal food demand, sustainable marketing strategy, OBOR, food quality certification

## Abstract

Muslim consumers prioritize the assurance of halal certification when purchasing products for consumption, as adherence to their religious beliefs is paramount. Within the framework of the One Belt One Road (OBOR) initiative, which encompasses approximately 72 countries—nearly half of which are predominantly Muslim—it is essential to recognize the significance of product integrity and authenticity. Given the substantial market potential in these regions, stakeholders must focus on meeting the demand for nutritional halal foods. This approach not only satisfies consumers’ religious requirements but also fosters trust and loyalty within this diverse demographic. An online survey consisting of two segments was conducted, and responses were analyzed using STATA version 16. The survey was disseminated through email, Facebook, and WhatsApp, resulting in a total of 435 responses, of which 399 were deemed complete and suitable for analysis. In the second segment, 279 respondents were evaluated, comprising 18 producers, 33 intermediaries, 70 wholesalers, and 158 retailers, to assess the growing market demand for nutritional halal foods and understand prevailing business trends. The results reveal that a 1-unit increase in the availability of product video links leads to a 63.4% increase in customer demand, while a 1-unit increase in customer belief and satisfaction results in increases of 23.3 and 5% in demand, respectively, highlighting the importance of transparency, religious compliance, and consumer trust in driving demand for halal products. The initial analysis of consumer behavior in OBOR Muslim countries, followed by a thorough examination of producers, intermediaries, wholesalers, and retailers, provides valuable insights into the projected growth in demand for Halal food. With an estimated 1.9 billion Muslims constituting approximately 25.01% of the global population—and 92% residing in OBOR nations—it is evident that halal dietary principles transcend national boundaries. By implementing strategic policies, stakeholders, including policymakers and firm managers, can optimize their efforts to meet the nutritional demands of this expansive market. Furthermore, this study identifies the need for additional exploration into related aspects such as sustainable traceability of quality halal products, brand image clarity, and consumer awareness of supply chain operations.

## 1 Introduction

The growing global Muslim population is poised to significantly impact supply chain operations and organizational practices, primarily due to the increasing prominence of the “halal” concept. Halal integrity, defined as consistent adherence to Islamic law throughout the entire supply chain of a product or service, is gaining traction not only within Muslim-majority regions but also in non-Muslim territories (1–3). Multinational corporations are increasingly recognizing the importance of Halal certification and the establishment of compliant supply chain operation standards to cater to this expanding market. This trend suggests a future where halal principles are not merely regional considerations but integral components of global supply chain strategies, demanding adaptation and innovation from organizations worldwide to ensure product integrity and meet diverse consumer needs (4, 5). The concept of “halal” has gained significant recognition and progress within various market environments. Currently, the global halal products market is estimated to be valued at approximately USD 2.1 trillion, highlighting the substantial economic impact of this sector. In response to the demands of Muslim consumers—the world’s second-largest religious demographic—there is a growing emphasis on ensuring that products strictly adhere to Islamic principles and regulations. This commitment has fostered significant growth in the global halal market, characterized by exceptional advancements in sustainable supply chain practices and product handling (6–8).

In the context of globalization, the production and distribution of products involve multiple sustainable operations and various stages before reaching the end consumer (9–11). However, consumers often lack awareness of the manufacturing processes and specific ingredients utilized, which can lead to uncertainty and skepticism regarding the quality and safety of food products. This lack of transparency ultimately undermines consumer trust and raises questions about the reliability of the products they consume. For Muslim consumers, the evaluation of a product’s healthfulness, safety, and hygiene is intrinsically linked to its adherence to the principles of clean handling and the use of quality ingredients. Islamic regulations stipulate that product ingredients must conform to specific religious standards, necessitating consumer awareness of these components. This understanding plays a critical role in influencing purchasing decisions, ultimately determining whether a product aligns with or contradicts religious beliefs. The significance of product ingredients is vital in shaping Muslim consumers’ judgments regarding the selection of quality products. It is widely acknowledged that consumers, particularly those who prioritize dietary restrictions and ethical considerations, exhibit heightened awareness and concern for the contents of their consumption. Such awareness is often fostered through accurate and transparent labeling, as highlighted in the works of Wang (12) and Yu et al. (13). Consequently, the clarity of ingredient information is essential in encouraging informed consumer choices within this demographic.

Precise food labeling is essential for ensuring food safety and effective quality management throughout the supply chain. Such labeling not only empowers consumers to make informed choices but also serves as a critical safeguard against both unintentional contaminants and economically motivated adulteration (EMA), which can introduce harmful toxins, pathogens, or allergens into food products (14, 15). By enforcing stringent labeling standards, stakeholders can enhance public health and strengthen trust in the food supply. Food originality is a paramount concern for customers, regulatory authorities, manufacturers, and supply chain operators, as inaccurate product labeling and various fraudulent activities can severely undermine consumer trust and safety. The implications of these discrepancies extend beyond individual transactions, potentially harming the integrity of the broader market and compromising the protection of the end consumer (16, 17). This study raises important questions regarding the objectives of this paper: (i) Does the video source link significantly affect the enhancement and sustainability of halal foods customer demand? (ii) Do independent variables such as customer belief, customer satisfaction, review mechanisms, customer perception, and customer intention significantly affect customer demand? (iii) Do moderating variables such as customer traceability, certification, and customer empowerment significantly impact the relationship between video source links and customer demand? (iv) Are investigations of producers, wholesalers, and retailers useful for estimating the average increase in demand for quality products in the market?

Consequently, the primary objective of this study is to emphasize the continuing and escalating demand for high-quality products, specifically in the food sector. This growth is not only anticipated but it is also considered essential for meeting the evolving needs of consumers. As societal standards for quality and safety in food rise, it is imperative for stakeholders to adapt and innovate to effectively meet these demands. The philosophy guiding this study aims to achieve a nuanced understanding of how the availability of product video sources influences the demand for halal foods, particularly within the context of the largest markets. By examining empirical evidence, this research strives to clarify the relationship between visual marketing strategies and consumer behavior, thereby contributing to the broader discourse on food consumption patterns in an increasingly diverse societal landscape. This study aims to elucidate various factors influencing the quality assurance, hygiene, traceability, and safety of food products. It provides a comprehensive analysis of the role and effectiveness of safety certification agencies globally, with a particular emphasis on their operations within China. Through this examination, the study deepens understanding of the mechanisms that ensure food safety and their implications for public health.

### 1.1 Theoretical background

Quality-certified products play a pivotal role in fostering confidence and trust among consumers regarding the religious compliance of food items. To achieve an unblemished and high-quality food supply chain, all producers, from suppliers to consumers, must uphold transparency and integrity within their operations, as consumers increasingly seek assurance regarding the honesty of the products they purchase (18). Consequently, maintaining rigorous standards of honesty presents a significant challenge for all entities involved in the supply chain (19, 20). Such products, which adhere to specific religious guidelines for quality, must embody the principles of tayyib—being both clean and of high quality for human consumption (21). The rising demand for quality products has subsequently enhanced confidence in safety certifications among both Muslim and non-Muslim consumers. Interestingly, the perception of quality is increasingly valued beyond religious considerations, positioning these standards as universal measures of product excellence (22).

The management of quality products and services is essential not only for business success but also for ensuring safe and beneficial consumption. In this context, the halal concept has gained significant importance in modern business practices. Studies demonstrate that the concept of halal transcends mere religious compliance, serving as a powerful framework for quality assurance that influences consumer attitudes, preferences, and values [(10, 23); Khan and Haleem, 2016]. Realizing halal’s potential requires comprehensive and unwavering integrity throughout the entire supply chain. This necessitates a meticulous approach covering all stages, from the initial procurement of raw materials to final product consumption. Production processes, packaging methods, accurate and transparent labeling, logistical considerations, retail practices, and, ultimately, consumer interaction must comply with established halal standards. Upholding ethical standards within this intricate network is not merely a compliance issue but a foundational principle that builds trust ensures consumer well-being, and ultimately strengthens the halal concept as a benchmark for quality. In conclusion, pursuing halal integrity is intrinsically linked to seeking quality, security, and ethical business practices in the modern marketplace. Ensuring high-quality assurance for the end consumer is crucial for enhancing perceptions of value and integrity (24–26). Therefore, effective management and assurance of halal integrity provide a comprehensive framework for quality assurance within the supply chain context, supported by various theoretical perspectives on operations management (27, 28). Thus, to ensure product quality, an honest guarantee framework is required to influence food composition, from preparation, slaughtering, and ingredients used to cleaning, handling, and processing, down to transportation and distribution, ultimately certifying that a product meets quality standards (29). Consequently, the development of Muslim consumers’ knowledge about their religion has become more specific regarding the types of products and services they use and consume, as shown in Table 1 studies. Manufacturers and suppliers use honest guarantees, marked with a certified logo, to inform and reassure their selected consumers that their products comply with halal and religious principles (30).

**Table 1:** Comparison with other recent studies.

Research papers	Halal products/foods	Food quality	Certification source	Video link source
Ali et al. (66)	✓	✓	✓	
Herliana and Zulfa (41)	✓		✓	
Artadita and Lestari (58)	✓	✓	✓	
Zhang et al. (89)	✓	✓		
Jiang et al. (86)	✓	✓	✓	
Wu et al. (90)	✓		✓	
Our paper	✓	✓	✓	✓

## 2 Materials and methods

The initial segment of the questionnaire aimed to test the relationship among independent, dependent, and moderating variables through correlation and moderation analyses, relevant to understanding factors influencing the growth in demand for halal products within One Belt One Road (OBOR) countries. The analysis employed linear regression probability statistics with partial least squares structural equation modeling (PLS-SEM). A robust examination of correlations was conducted to facilitate the statistical evaluation of the data, utilizing STATA version 16 as the statistical analysis software. Hypotheses were initially formulated and subsequently tested, leading to the selection of mechanisms to establish the hypothesized structural model. All items in the hypothesis model were examined; secondly, to evaluate the structural model, ANOVA, correlation, and robust analysis were conducted to assess the sustainable growth of quality products. An online survey conducted via Facebook, WhatsApp, and email aimed to gather responses from consumers in OBOR Muslim countries included in the OBOR Initiative. A total of 35 items were selected for research purposes, including 30 related to various categories of sustainable factors affecting the growth of quality product variables in the research model and five items focusing on the sociodemographic characteristics of customers. Each research factor comprised three items, totaling 10 factors. The online survey achieved participation from 435 respondents, of whom 399 completed the questionnaire. All respondents remained anonymous and were adequately informed about the study’s content. The majority of participants held advanced degrees, including bachelor’s, master’s, and PhD qualifications, and possessed relevant experience in purchasing certified products. The survey included various closed-ended questions addressing nine significant variables, analyzed using a 5-point Likert scale ranging from strongly disagree to strongly agree. The choice of sampling technique was a crucial aspect of the data collection methodology. In the second part of the questionnaire, producers, intermediaries, wholesalers, and retailers were investigated to examine the percentage increase in higher-quality food demand in their marketplaces. In this section, 18 producers, 33 intermediaries, 70 wholesalers, and 158 retailers were surveyed to determine the estimated rise in the nutritional demand for halal foods in the market, resulting in a total of 279 respondents to evaluate business market trends.

### 2.1 Study framework

In the initial stage of this study, we present conceptual insights related to the OBOR initiative, accompanied by a link to a product video source. This initiative highlights the significant proportion of Muslims residing in OBOR countries, positioning this region as a burgeoning halal food market. Chinese food companies can capitalize on this opportunity by building trust and confidence among Muslim consumers through the use of product video links, thereby enhancing food safety and quality assurance. By cultivating a favorable market environment, these firms can secure sustainable demand for their certified products. The OBOR initiative represents China’s commitment to enhancing connectivity and cooperation among nations, revitalizing ancient trade routes, and attracting global attention to participating countries. Furthermore, the OBOR initiative supports the establishment of a reliable supply chain network, ensuring timely food delivery while promoting infrastructure development, trade, and investment across the region (31).

**Figure 1:**
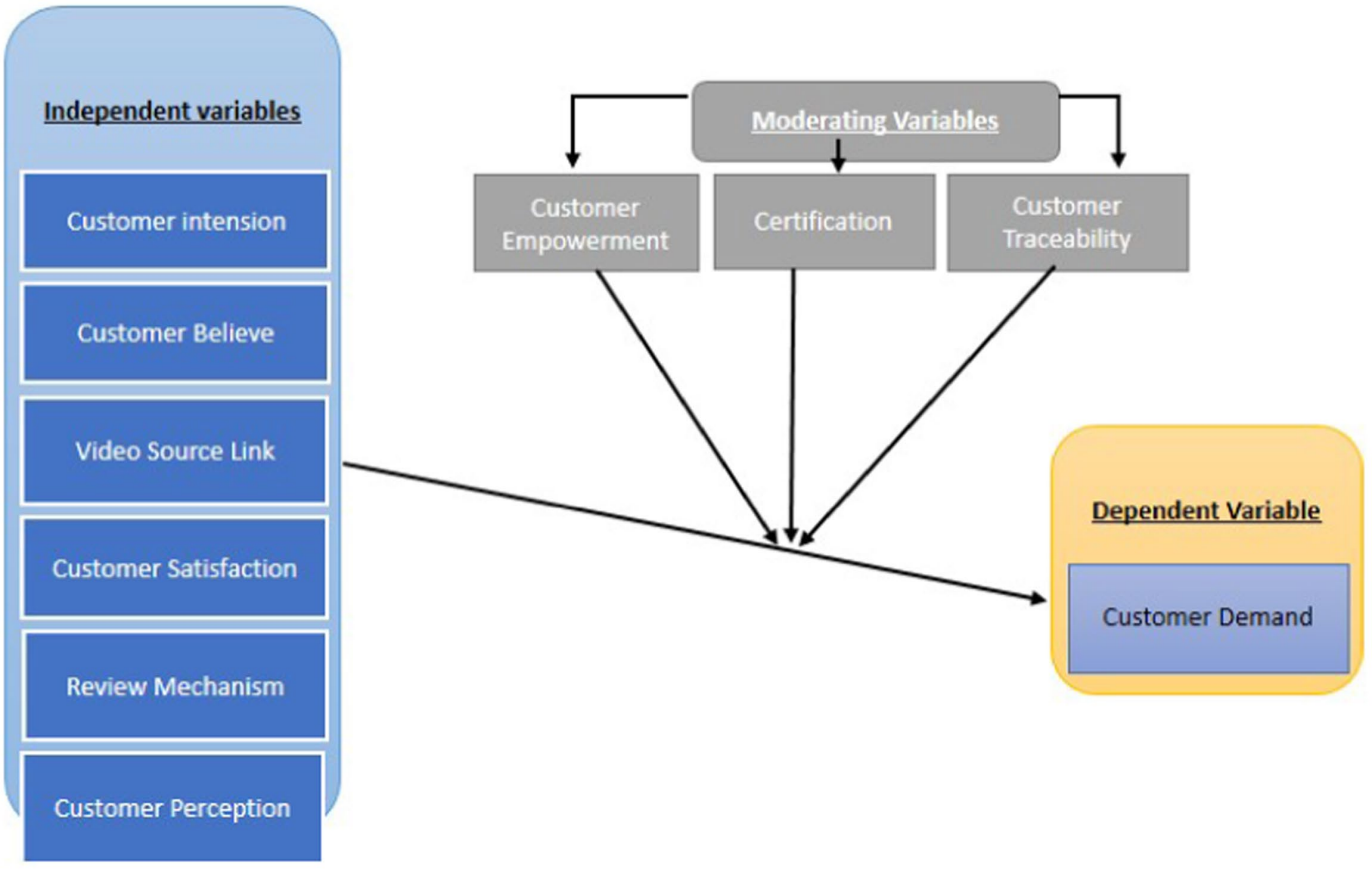
Graphical research model.

The graphical model presented in Figure 1 illustrates the dynamic relationship between the independent variable of product video source links and the dependent variable of customer demand, alongside other independent and moderating variables. This model emphasizes the pivotal role that transparency plays in influencing Muslim consumers’ trust in halal food products. By providing verifiable information regarding compliance with halal principles via websites and mobile applications, consumers are better equipped to make informed purchasing decisions. Nonetheless, various challenges, misconceptions, and supply chain issues hinder the holistic implementation of halal practices. Ensuring high-quality food production demands a comprehensive understanding of supply chain sustainability, which is crucial for maintaining the integrity of certified products. Many suppliers and manufacturers lack the necessary awareness of the importance of adhering to certified food processes, leading to concerns among Muslim consumers about the quality, hygiene, and safety of goods in line with Islamic teachings.

In the context of this research model, customer perception, intention, belief, satisfaction, and review mechanisms are identified as independent variables, each demonstrating a significant relationship with a dependent variable. This framework draws on existing studies in the field, which elucidate the roles of these variables in shaping customer demand. Notably, in local markets characterized by limited supply chain activities, consumers often rely on distinct beliefs when selecting products, aligning their choices with certified concepts (32, 33). In contrast, from a global market perspective, consumers must adhere to established concepts based on the information available on product packaging (34, 35), including certified logos and integrity. According to Van der Lans et al. (36), trust is a predictive measure of the company’s ability to consistently meet consumer beliefs and avoid causing harm to customers. Therefore, independent variables are crucial in maintaining and enhancing the positive relationship between customer demand and traceable sources from companies regarding products, ultimately contributing to a positive market image. Customer empowerment, certification, and traceability are considered moderating variables because they influence the relationship between traceable variables and customer demand, as supported by previous studies on quality food traceability sources and customer demand. Several businesses have emerged that utilize misleading statements and certified logos related to their products and services (37–39).

The effect of these moderating variables, derived from several previous studies, indicates a significant positive relationship between independent and dependent variables. However, numerous publications highlight that these moderating variables also show a strong positive effect between independent and dependent variables. Customer demand increases due to the influence of product video sources, which effectively convey production series information to customers. Producers play a crucial role as they seek to understand the entire supply chain and production processes. In the pursuit of transparency, companies must provide comprehensive information regarding their practices and procedures, thereby ensuring clarity for consumers while protecting sensitive data from competitors. This vigilance is particularly important regarding non-certified products and ingredients, which pose a risk to the integrity of halal certification (40, 41). It is crucial to recognize that halal certification transcends mere compliance with Muslim standards; it represents a global commitment to quality and ethical living (42, 43). Furthermore, the halal status of products is intricately linked to the risk of contamination with haram elements during logistics and transportation processes (3). The perception of Muslim customers as a potential threat to certified products raises significant concerns within the food industry. Despite the importance of ensuring quality food perceptions and maintaining product quality through safe supply chain transportation, empirical evidence suggests that not all multinational food companies practice transparent supply chain methods. Maintaining product quality requires honesty across various aspects, including staffing, raw materials, production processes, transportation, and storage. Consequently, leading food corporations, such as McDonald’s and Nestlé, are increasingly committed to ensuring their products meet certification standards, particularly in Muslim-majority markets (44, 45).

### 2.2 Study conceptual model

**Figure 2:**
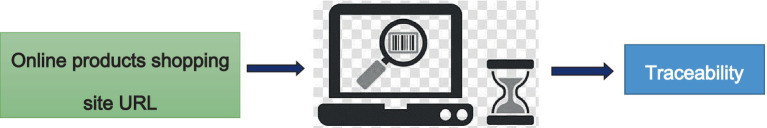
Study conceptual model “Product Video Source Link”.

The product video source link, titled “A product video source link is the complete production process information of a product regarding its manufacturing through a short video clip provided on the product envelope,” is shown in Figure 2. Customers can verify the certified information by searching for this link on various internet search engines. When searching the video source link, a specific clip provides production details about the quality foods of a particular product, which enhances the assurance of certified products in the minds of Muslim customers. This video source link increases satisfaction levels among Muslim consumers, encouraging them to purchase more from Chinese food firms. It also heightens the psychological impact on Muslim consumers regarding their purchasing decisions by providing this additional feature related to traceability. The video source link presents transparent background information about the food manufacturing process, ensuring that consumers have access to tracing data and can observe the actual food production conditions (46). In an era dominated by advanced technology, consumers predominantly prefer to use search options to understand the origins of their food. The video source link enhances consumer satisfaction through visual advertisements. Chinese food firms can build sustainable confidence among Muslim consumers by utilizing video source links, leading to a significant market share in the global halal products industry.

Several food economists project that the halal food industry is poised to become a dominant market force in the near future, driven by four significant trends. First, Islam is the fastest-growing religion globally, fostering consistent demand for quality halal products, with annual consumption increases estimated at 16%. Second, a notable shift among non-Muslim consumers toward halal products is emerging, driven by concerns about quality and safety; for instance, in the UK, while there are approximately 2 million Muslims, the total consumer base for halal meat is estimated to be around 6 million. Third, the rise of high-quality consumer goods correlates with the expanding Muslim population and their increasing disposable income (47, 48). Finally, there is a growing awareness among Muslims regarding the importance of certified halal products, as evidenced by remarkable annual sales growth of quality foods across Europe, with demand surging between 30 and 40%.

## 3 Results

Recent findings indicate a significant correlation between the presence of video source links for products and the demand among Muslim consumers for high-quality goods. As these consumers engage with video content, their purchase intentions and trust levels significantly increase, leading to an enhanced readiness to make immediate purchases. The incorporation of traceability, certification, and customer empowerment mechanisms further bolsters their confidence in the featured products. Specifically, video clips provide crucial information that aligns with consumer specifications and preferences, catering to regional market demands. Consequently, as engagement with product-related videos increases, so does consumer demand, driven by heightened trust and satisfaction in the assurance of product quality. This relationship highlights the positive impact of visual content on consumer behavior in the context of market dynamics (49).

In response to growing consumer concerns regarding food safety and traceability, the OBOR initiative presents an essential framework for enhancing food logistics. By facilitating a reliable and efficient transportation system, the OBOR initiative can strengthen regulatory support to ensure the safety and quality of food products. This is particularly crucial in addressing the increasing demand for food, as well-established global logistics networks enable companies to engage in cost-effective international trade without compromising quality and quantity standards. Therefore, it is imperative that regulations evolve in tandem with these systems to reinforce consumer confidence in food safety. According to prior statistics, Muslims present the largest international industry for certified products, with their purchasing decisions strongly influenced by religious obligations and ethical considerations. This commitment to authenticity and integrity extends to all aspects of Muslim life, particularly regarding product quality and certification. As a result, the market for certified products presents a vast opportunity (50, 51). To fully capitalize on this potential, businesses—including those owned by non-Muslim entrepreneurs—must take into account key quality attributes that align with consumer expectations. Future projections highlight that product certification plays a pivotal role in sustaining business competitiveness and ensuring long-term success in this highly favorable and profitable market (52).

The statistical analysis of the research questionnaire used for this study is shown in Table 2. The questionnaire consists of 35 items, with data collected from 399 respondents. The statistical results for each item are satisfactory. Of the 35 items, 30 were developed to measure independent variables, dependent variables, and moderating variables, while the remaining five items focused on social demographic factors. The statistical analysis includes mean, standard deviation, minimum, maximum, variance, skewness, and kurtosis for each item, all of which align satisfactorily with the hypotheses formulated in this study on the demand for halal products. Certified supply chain management involves overseeing the distribution of demanded products from several suppliers to various end-customer nodes, encompassing multiple stakeholders positioned at different points in the supply chain. These stakeholders may also be involved in managing non-certified products to meet the needs of both certified and non-certified product customers (53). The certified supply chain distinguishes itself from the standard supply chain by prioritizing not only customer satisfaction but also product integrity throughout the entire supply chain process. This approach ensures that certified products, which are now manufactured across various regions worldwide, maintain their quality and authenticity until reaching the consumer (54). Maintaining the quality attributes of certified foods is paramount and requires vigilant coordination among all parties involved. In light of growing global concerns regarding food safety and security, particularly in relation to disease prevention, compelling evidence suggests that non-Muslim consumers are increasingly inclined toward high-quality food options that prioritize safety, hygiene, and quality assurance (55–57). This shift in consumer preferences reflects a broader demand for certified products, which are characterized by stringent ingredient requirements that minimize contamination risks. As a result, the market for certified goods is expanding beyond traditional practices, encompassing a diverse array of certified food items, lifestyle choices, and related services (58).

**Table 2:** Statistical analysis of the research questionnaire.

Questions	*N*	Range	Minimum	Maximum	Mean		Std. Deviation	Variance	Skewness		Kurtosis	
	Statistic	Statistic	Statistic	Statistic	Statistic	Std. Error	Statistic	Statistic	Statistic	Std. Error	Statistic	Std. Error
Customer responses	400	399.00	1.00	400.00	200.5000	5.78072	115.61430	13366.667	0.000	0.122	−1.200	0.243
Perception (Q1)	400	4.000	1.000	5.000	3.79500	0.063285	1.265693	1.602	−1.070	0.122	0.050	0.243
Perception (Q2)	400	4.00	1.00	5.00	4.1250	0.04476	0.89520	0.801	−1.428	0.122	2.663	0.243
Perception (Q3)	400	4.00	1.00	5.00	4.1675	0.04434	0.88680	0.786	−1.786	0.122	4.346	0.243
Satisfaction (Q1)	400	4.00	1.00	5.00	4.2475	0.04056	0.81110	0.658	−1.501	0.122	3.325	0.243
Satisfaction (Q2)	400	4.00	1.00	5.00	4.1525	0.04448	0.88951	0.791	−1.270	0.122	1.875	0.243
Satisfaction (Q3)	400	4.00	1.00	5.00	4.1525	0.04377	0.87531	0.766	−1.203	0.122	1.708	0.243
Traceability (Q1)	400	4.00	1.00	5.00	4.1550	0.04008	0.80161	0.643	−1.167	0.122	2.109	0.243
Traceability (Q2)	400	4.00	1.00	5.00	4.0325	0.04510	0.90193	0.813	−1.341	0.122	2.382	0.243
Traceability (Q3)	400	4.00	1.00	5.00	4.0875	0.04683	0.93650	0.877	−1.371	0.122	2.063	0.243
Mechanism (Q1)	400	4.00	1.00	5.00	4.2150	0.04140	0.82794	0.685	−1.431	0.122	2.893	0.243
Mechanism (Q2)	400	4.00	1.00	5.00	4.1075	0.04480	0.89608	0.803	−1.326	0.122	2.177	0.243
Mechanism (Q3)	400	4.00	1.00	5.00	4.1325	0.04335	0.86708	0.752	−1.280	0.122	2.139	0.243
Certification (Q1)	399	4.00	1.00	5.00	4.2155	0.04569	0.91259	0.833	−1.616	0.122	3.126	0.244
Certification (Q2)	400	4.00	1.00	5.00	4.2575	0.04162	0.83242	0.693	−1.587	0.122	3.471	0.243
Certification (Q3)	400	4.00	1.00	5.00	4.2850	0.04109	0.82187	0.675	−1.523	0.122	3.193	0.243
Empowerment (Q1)	400	4.00	1.00	5.00	4.2350	0.04866	0.97321	0.947	−1.633	0.122	2.614	0.243
Empowerment (Q2)	400	4.00	1.00	5.00	4.1850	0.04522	0.90433	0.818	−1.518	0.122	2.738	0.243
Empowerment (Q3)	400	4.00	1.00	5.00	4.1750	0.04462	0.89239	0.796	−1.372	0.122	2.137	0.243
Intension (Q1)	400	4.00	1.00	5.00	4.1525	0.04391	0.87816	0.771	−1.418	0.122	2.532	0.243
Intension (Q2)	400	4.00	1.00	5.00	4.2075	0.04202	0.84036	0.706	−1.477	0.122	3.029	0.243
Intension (Q3)	400	4.00	1.00	5.00	4.3100	0.04033	0.80655	0.651	−1.456	0.122	2.777	0.243
Believe (Q1)	400	4.00	1.00	5.00	4.2600	0.04284	0.85688	0.734	−1.560	0.122	3.171	0.243
Believe (Q2)	400	4.00	1.00	5.00	4.3200	0.04429	0.88575	0.785	−1.847	0.122	4.094	0.243
Believe (Q3)	400	4.00	1.00	5.00	4.3325	0.04483	0.89663	0.804	−1.774	0.122	3.569	0.243
Demand (Q1)	400	4.00	1.00	5.00	4.3250	0.03956	0.79116	0.626	−1.471	0.122	3.142	0.243
Demand (Q2)	400	4.00	1.00	5.00	4.2550	0.04233	0.84662	0.717	−1.583	0.122	3.394	0.243
Demand (Q3)	400	4.00	1.00	5.00	4.3775	0.03763	0.75260	0.566	−1.598	0.122	3.909	0.243
Video link (Q1)	400	4.00	1.00	5.00	4.3550	0.03760	0.75192	0.565	−1.755	0.122	5.124	0.243
Video link (Q2)	400	4.00	1.00	5.00	4.3275	0.03505	0.70105	0.491	−1.519	0.122	5.028	0.243
Video link (Q3)	400	4.00	1.00	5.00	4.4200	0.03463	0.69268	0.480	−1.598	0.122	4.859	0.243
Gender	400	1.00	1.00	2.00	1.2950	0.02283	0.45661	0.208	0.902	0.122	−1.192	0.243
Age	400	2.00	1.00	3.00	1.6975	0.03029	0.60573	0.367	0.260	0.122	−0.622	0.243
Marital status	400	2.00	1.00	3.00	1.4000	0.02478	0.49559	0.246	0.472	0.122	−1.622	0.243
Income	400	2.00	1.00	3.00	1.3675	0.02798	0.55967	0.313	1.226	0.122	0.529	0.243
Education	400	3.00	1.00	4.00	1.9550	0.04014	0.80286	0.645	0.870	0.122	0.719	0.243
Valid N (Listwise)	399											

The sociodemographic characteristics of the study’s participants reveal significant trends among the respondent population. Notably, individuals aged 25 to 35 comprised 91.92% of the sample, indicating a strong interest in the study’s themes. The number of male respondents exceeded that of female respondents, with a ratio of 70.92%. Furthermore, consumers with an income between $100 and $1,000 demonstrated a pronounced preference for high-quality products, accounting for 95.98% of responses. Marital status analysis indicated that married Muslim consumers showed greater demand for halal products, comprising 61.40% of the sample. Additionally, the majority of participants held advanced degrees, specifically master’s and PhD qualifications, representing 66.91% of the sample, as detailed in Table 3. The distribution of respondents across various roles—producers, intermediaries, wholesalers, and retailers—was recorded at 6.45, 11.82, 25.08, and 56.63%, respectively, in the second part of the questionnaire.

**Table 3:** Sociodemographic sample characteristics.

Variable	Description	Number of observations	Percent %
Gender	Men	283	70.92
	Women	116	29.07
Age	Less than 25 years	148	37.09
	Between 25 to 30 years	216	54.13
	More than 35	35	8.77
Education	Bachelor’s degree	107	26.81
	Master’s degree	229	57.39
	PhD	38	9.52
	Others	35	8.77
Household income (USD per month)	$100USD to $500USD	269	67.41
	$500USD to $1000USD	114	28.57
	Above $1000USD	16	4.01
Marital status	Single	245	61.40
	Married	154	38.59
	Producers	18	6.45
Actors	Intermediaries	33	11.82
	Wholesalers	70	25.08
	Retailers	158	56.63

Table 4 illustrates the positive correlations among independent, dependent, and moderating variables, indicating a favorable increase in demand for halal products. All variables demonstrate significant interrelations, particularly in relation to the percentage of Muslim consumers engaging with video clips, which in turn stimulates demand for certified products. The provision of a video source link enhances Muslim consumers’ purchase intentions, beliefs, trust, confidence, and satisfaction, thereby promoting immediate readiness to pay. Furthermore, these video clips serve to inform consumers, amplifying demand for halal-certified products in accordance with customer specifications and market pressures. The emphasis on high-quality products during the consumer knowledge development phase is critical for fostering demand, as highlighted by Adams et al. (59) and Sahir et al. (60). Ultimately, increased access to video source links correlates directly with heightened product demand.

**Table 4:** Correlation among variables.

Correlation	Customer demand	Customer perception	Customer satisfaction	Review mechanism	Customer intension	Customer belief	Video link source	Certification	Customer traceability	Empowerment
Demand	1									
Perception	0.363^***^	1								
Satisfaction	0.404^***^	0.484^***^	1							
Mechanism	0.473^***^	0.327^***^	0.418^***^	1						
Intension	0.486^***^	0.283^***^	0.377^***^	0.446^***^	1					
Belief	0.598^***^	0.337^***^	0.377^***^	0.438^***^	0.583^***^	1				
Video link	0.756^***^	0.362^***^	0.446^***^	0.521^***^	0.539^***^	0.546^***^	1			
Certification	0.419^***^	0.347^***^	0.470^***^	0.738^***^	0.419^***^	0.383^***^	0.497^***^	1		
Traceability	0.449^***^	0.418^***^	0.435^***^	0.623^***^	0.339^***^	0.393^***^	0.464^***^	0.518^***^	1	
Empowerment	0.456^***^	0.355^***^	0.502^***^	0.441^***^	0.587^***^	0.479^***^	0.477^***^	0.539^***^	0.504^***^	1

^*^p < 0.05, ^**^p < 0.01, ^***^p < 0.001.

**Figure 3:**
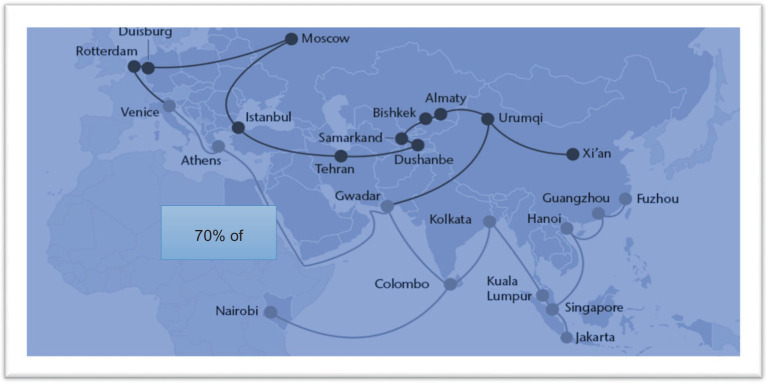
OBOR continents area and halal products supply chain routes.

The delivery of quality products to several continents is made possible by highlighted supply chain routes that cover 70% of the global land area, as demonstrated in the figure above, which includes land, sea, and air routes presented in Figure 3. The relationship ratios of individual variables show a moderating impact, such as video link (0.64), customer belief (0.24), review mechanism (0.044), customer satisfaction (0.011), and customer perception (0.044), all of which have positive and significant relationships with customer demand (0.18). The Cronbach’s alpha value is greater than 0.70, indicating that the scale is reliable. This study comprises 30 items designed to assess the demand among Muslim consumers for certified products from Chinese food firms. The ANOVA results indicate significant differences in consumer perceptions regarding the demand for certified food, as well as in the assessments of producers, wholesalers, and retailers concerning the surge in demand for higher-quality food in specific regions, as evidenced in Tables 5, 6. Notably, these items exhibit a strong correlation with an average reliability coefficient of 0.935, signaling high interrelatedness. Previous research has largely focused on consumer perceptions of food traceability systems, which are a crucial aspect of purchasing behavior. The empowerment of consumers through the extensive use of informational resources across various lifestyle aspects is closely linked to the evolution of information technology, reflecting an inherent demand for quality-driven products and services (10, 61–63).

**Table 5:** ANOVA test results comparing average differences among consumers regarding halal food.

	Sum of squares	Df	Mean Squares	F	Sig.
Between groups	0.005	2	0.002	2.217	0.077
Within groups	0.394	397	0.001		
Total	0.399	399			

**Table 6:** ANOVA test results comparing the average differences among producers, wholesalers, and retailers regarding halal food.

	Sum of squares	Df	Mean squares	F	Sig.
Between groups	131.584	4	51.098	23.590	0.000
Within groups	147.966	275	2.180		
Total	279.550	279			

The demonstration of the regression analysis for this study is presented in Table 7. The trend of the findings is consistent with the current outcomes, indicating that the primary independent variable, the impact of the video source link, is statistically significant for customer demand for quality food [Coef. = 0.634; *p*-value = 0.11]. Similarly, the effect of customer beliefs is also statistically positive and significant in relation to customer demand [Coef. = 0.233; *p*-value = 0.15], while the impacts of the other independent variables on customer demand are positive and statistically significant. Additionally, the effects of the moderating variables are statistically positive and significant between the video link and customer demand; specifically, one moderating variable, Traceability, shows an effect [Coef. = 0.047; *p*-value = 0.27], while the remaining moderating impacts among independent and dependent variables are significant and positive. The importance of consuming certified products is underscored by the religious and moral obligations guiding Muslims, regardless of their context, whether at home or abroad (64, 65). This adherence to faith-based dietary laws suggests that an individual’s perception of religious responsibilities significantly influences their inclination to procure certified food items. Consequently, the element of belief emerges as a critical factor, potentially exerting greater significance in the context of certified products compared to non-certified alternatives (66, 67).

**Table 7:** OLS regression analysis.

Measurement item	Coef.	St.Err.	*t*-value	*p*-value	[95% Conf Interval]	Sig
Perception	0.037	0.031	1.20	0.232	−0.024	0.097
Satisfaction	0.005	0.035	0.13	0.893	−0.065	0.074
Review mechanism	0.05	0.051	1.00	0.32	−0.049	0.15
Customer intension	−0.02	0.044	−0.44	0.658	−0.107	0.067
Customer belief	0.233	0.041	5.68	0.011	0.152	0.313***
Video source link	0.634	0.047	13.55	0.015	0.542	0.726***
Certification	−0.052	0.048	−1.07	0.284	−0.147	0.043
Customer traceability	0.047	0.043	1.10	0.27	−0.037	0.131
Customer empowerment	0.039	0.041	0.96	0.34	−0.042	0.12
Constant	0.116	0.179	0.65	0.516	−0.235	0.468
Mean dependent var	4.320		SD dependent var.		0.693	
R-squared	0.629		Number of obs		399.000	
F-test	73.161		Prob > F		0.000	
Akaike crit. (AIC)	463.698		Bayesian crit. (BIC)		503.588	

***p < 0.01, **p < 0.05, *p < 0.1.

The halal products industry presents an opportunity for firms to navigate the complexities of the certified products market, facilitating productive collaboration among various stakeholders, such as honest food suppliers, product producers, traders, warehousing service providers, regulatory authorities, and discerning customers. However, there remains a lack of a definitive framework addressing the global certified products market. For a nation to position itself as a potential leader in this global market, it must create a robust environment that ensures a sustainable supply chain in line with international quality standards, alongside effective regulatory oversight to maintain the guaranteed integrity of halal products (68–70).

The robust outcomes of regression analysis revealed consistent results within the path measurement framework, indicating a substantial and statistically significant positive effect of video links on customer demand for certified food, with a *p*-value of 0.000 (*p* < 0.01). Moreover, the influence of other independent variables also demonstrated statistical significance (*p* < 0.01), while moderating variables exhibited a notable positive impact on the relationship between video links and customer demand, as corroborated by data presented in Tables 8, 9. This analysis underscores the necessity for meticulous traceability in supply chain operations to prevent the infiltration of hazardous and non-certified materials, as established by Wan and Guo (87). Furthermore, existing literature (71–73) highlights the significant role of Muslim religious sentiments and beliefs surrounding the authenticity of certified products in shaping quality food demand choices.

**Table 8:** Variance inflation factor analysis.

Measurement item	VIF	1/VIF
Review mechanism	2.88	0.35
Certification	2.60	0.38
Customer empowerment	2.13	0.47
Customer intension	2.03	0.49
Customer traceability	1.99	0.50
Video link source	1.89	0.53
Customer belief	1.80	0.56
Customer satisfaction	1.68	0.59
Customer perception	1.45	0.69

**Table 9:** Robustness analysis: Model 1 and Model 2 presented without a moderator; Models 3, 4, and 5 presented with moderator effects.

Variables	Model 1	Model 2	Model 3	Model 4	Model 5
Perception	0.0438 (0.0299)	0.0369 (0.0301)	−0.0705 (0.116)	0.118 (0.161)	−0.0674 (0.116)
Satisfaction	0.0113 (0.0407)	0.00474 (0.0395)	0.323** (0.129)	0.300 (0.186)	0.253 (0.197)
Mechanism	0.0440 (0.0351)	0.0504 (0.0473)	0.205 (0.200)	−0.130 (0.192)	−0.138 (0.164)
Intension	−0.00877 (0.0463)	−0.0196 (0.0476)	−0.419* (0.217)	−0.00636 (0.0458)	0.0412 (0.216)
Believe	0.241*** (0.0553)	0.233*** (0.0559)	−0.198 (0.179)	−0.0535 (0.219)	−0.225 (0.190)
Video link	0.637*** (0.0491)	0.634*** (0.0496)	1.041*** (0.248)	0.803** (0.322)	1.027*** (0.288)
M1Traceablity			0.0281 (0.0290)		
M2Traceability			−0.0820** (0.0319)		
M3Traceability			−0.0409 (0.0469)		
M4Traceability			0.103** (0.0512)		
M5Traceability			0.112** (0.0452)		
M6Traceability			−0.103* (0.0600)		
Certification		−0.0519 (0.0456)			
Traceability		0.0470 (0.0377)			
Empowerment		0.0393 (0.0409)			
M1Certification				−0.0183 (0.0382)	
M2Certification				−0.0695 (0.0452)	
M3Certification				0.0528 (0.0463)	
M4Certification				0.0717 (0.0513)	
M5Certification				−0.0465 (0.0770)	
M6Certification				0.0000 (0.0000)	
M1Empowerment					0.0261 (0.0284)
M2Empowerment					−0.0629 (0.0453)
M3Empowerment					0.0469 (0.0408)
M4Empowerment					−0.0108 (0.0522)
M5Empowerment					−0.0994 (0.0684)
M6Empowerment					0.113** (0.0487)
Constant	0.130 (0.203)	0.116 (0.205)	0.217 (0.171)	0.0454 (0.248)	0.233 (0.187)
Observations	400	399	400	399	400
R-squared	0.625	0.629	0.642	0.631	0.640

Robust standard errors in parentheses ***p < 0.01, **p < 0.05, *p < 0.1.

**Figure 4:**
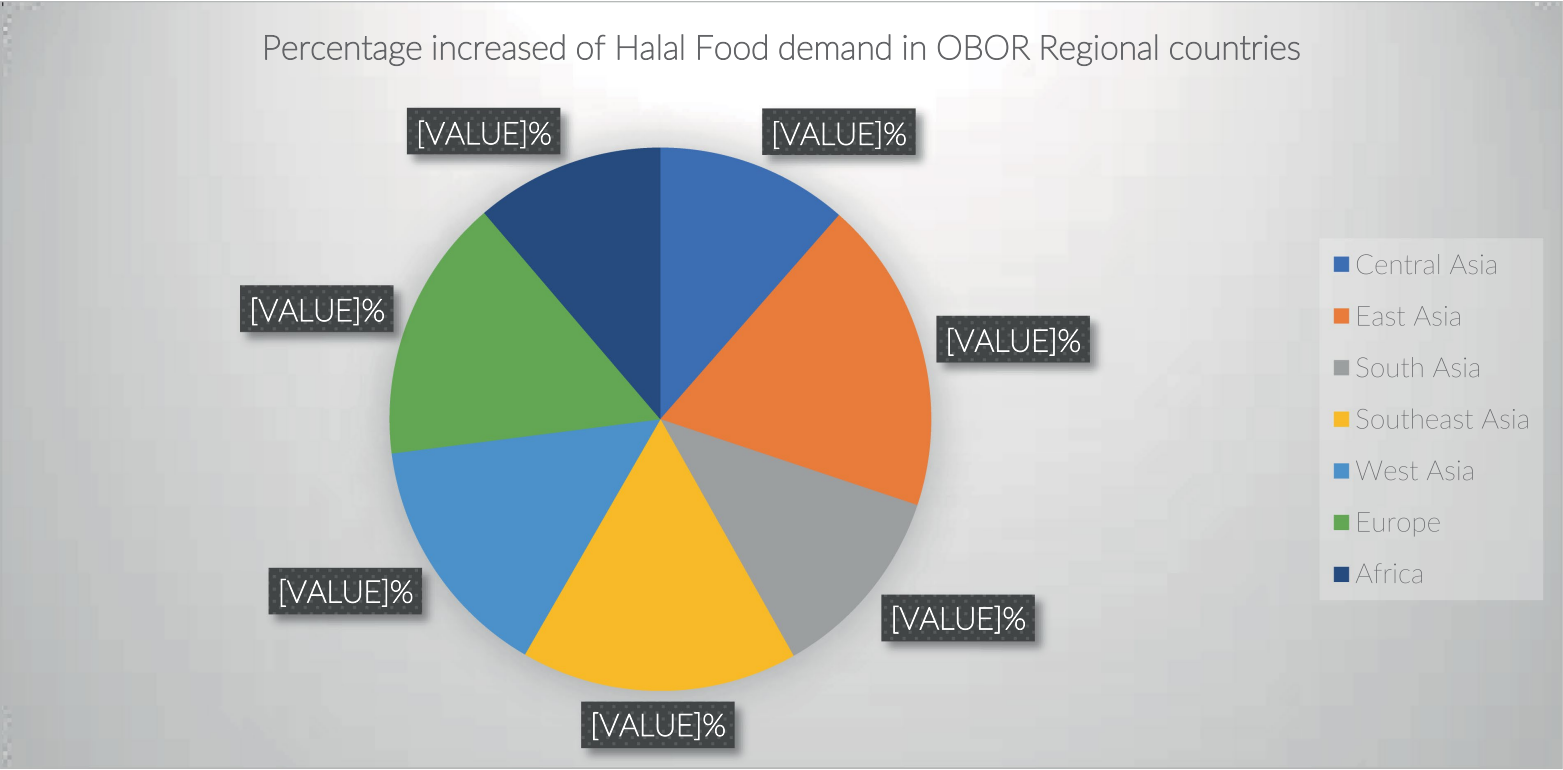
Percentage increase in demand for halal foods in OBOR countries across different regions.

The average demand increase across various regions included in the OBOR initiative reveals significant opportunities within the global halal market, valued at approximately $2.3 trillion USD, excluding Islamic finance. With an estimated annual growth rate of 20%, this market is projected to be worth $560 billion USD. The halal industry caters not only to the dietary preferences of the 1.8 billion Muslims worldwide but has also expanded to encompass a diverse range of sectors, including cosmetics, pharmaceuticals, health products, food items, and medical devices. Additionally, it has extended into the service sector, covering logistics, marketing, branding, and traceability. Muslims, constituting roughly 23% of the global population, represent a robust consumer base with a consistent growth rate of 3% per year. The halal certification system is essential for ensuring safety and quality across food chains, thereby bolstering consumer trust and confidence in these products (74, 75) (Figure 4).

**Figure 5:**
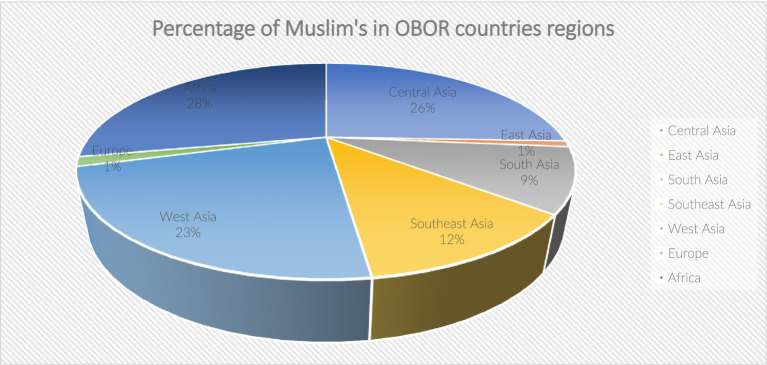
Average percentage of Muslims in countries of different OBOR regions.

The presence of variables exhibiting a Variance Inflation Factor (VIF) of 10 or higher, as indicated in Table 9, suggests significant collinearity among the assessed criteria. Notably, the dietary beliefs of Muslims remain consistent regardless of geographic location, as evidenced by the population percentages illustrated in Figure 5. Additionally, including product video source links enhances the trust levels of Muslim consumers regarding their purchase intentions for certified products from Chinese food firms. This development presents an opportunity for these firms to penetrate a larger market among regional consumers. Furthermore, the OBOR initiative, characterized by its advanced transportation network, facilitates improved traceability for certified products, enabling consumers in these regions to access products more readily through designated sales platforms. The implementation of the OBOR initiative presents a significant opportunity for Chinese firms to enter the expansive market of certified products, particularly within the Muslim demographic (76–78). This mega project can facilitate substantial access to these markets, fostering sustainable growth in food safety. The availability of short video clips online enhances consumer assurance regarding certified products by providing increased traceability. Such resources contribute to rising demand for quality products, as they bolster consumer confidence among Muslim consumers who value transparency in production processes. Consequently, it is imperative for management authorities to safeguard consumer interests by establishing effective review mechanisms that accurately assess the certification levels of food compositions and ingredients provided by suppliers (88).

## 4 Discussion

The OBOR initiative, emphasizing infrastructural development and enhanced connectivity, has inadvertently created a fertile ground for the proliferation and demand of certified products catering to Muslim consumers. This is primarily due to the initiative’s facilitation of a robust transportation network, drastically reducing logistical hurdles and enabling the efficient distribution of halal-certified goods across participating nations. Previously limited by geographical constraints and logistical complexities, access to a diverse range of compliant products has expanded significantly, boosting consumer confidence and fueling demand (79). This trust is further enhanced by the increasing prevalence of product video links accompanying these goods. These videos, often showcasing the sourcing, processing, and certification process, serve as crucial tools in assuring food safety and quality. They also provide a vital element of traceability, allowing consumers to verify the authenticity of certifications and gain insight into the product’s journey from origin to consumption (80).

In conclusion, the OBOR initiative, while primarily focused on infrastructural advancement, plays a significant role in fostering a more connected and trustworthy halal marketplace. The combination of improved transport networks and readily available product information, particularly through video links, empowers Muslim consumers to make informed choices, driving the demand for demonstrably certified and traceable products (81). This synergistic effect highlights the indirect yet profound impact of the OBOR initiative on the global halal economy. Recent trends in the Chinese food industry suggest a compelling interplay between digital engagement, supply chain management, and business sustainability. Chinese food companies are experiencing an increase in certified product sales, demonstrably linked to consumer satisfaction derived from visually engaging online video content showcasing product features. This heightened consumer confidence directly translates into increased demand for specific brands. Concurrently, the strategic management of the OBOR supply chain network plays a crucial role in ensuring food safety and reliability. This enhanced supply chain integrity, as highlighted by Monaghan (82) and Wang (83), further bolsters consumer trust and reinforces the demand for products sourced from affiliated food companies. Ultimately, this synergy—leveraging online visibility to build trust in a robust and reliable supply chain—significantly contributes to the long-term sustainability of these businesses. The ability to transparently showcase product features and assure consumers of safety through optimized supply chain practices positions these Chinese food companies for continued growth and market leadership (2).

The data regarding the percentage of Muslim customers from OBOR countries is taken from Google, which is a reliable source. The OBOR initiative includes 72 countries where the number of Muslim consumers is significant, as shown in Table 10. These countries are situated in East Asia, Southeast Asia, South Asia, Central Asia, the Middle East, North Africa, and Europe, along with some nations included via the 21st Century Maritime Silk Road. Therefore, while Muslim consumers are found all over the world, the majority of their large population is located in OBOR countries. Many Muslim countries within the OBOR initiative have more than 90% of their population as Muslims, and the concept of certified food is significant in every region of these countries. Additionally, they often prefer not to discuss haram foods due to their religious beliefs. The terms halal and haram have opposing meanings, indicating that Muslim consumers seek to purchase higher-quality products that align with their religious beliefs. Every Muslim consumer strives to ensure that the products they intend to buy for consumption are certified. If Chinese food firms include a link to a product video source, it can enhance the trust of Muslim consumers and increase their demand for certified products from these firms. The OBOR initiative facilitates the delivery of products to several countries through different transportation channels, all of which require maintaining product quality assurance for customers (41, 85).

**Table 10:** Percentage of Muslim consumers in the OBOR initiative.

Countries Name	Muslim %	Countries Name	Muslim %	Countries Name	Muslim%	Countries Name	Muslim %	Countries Name	Muslim %	Countries Name	Muslim %
China	2.8	Malaysia	61.3	Iraq	95	Herzegovina	51	Singapore	15	Syria	60
Russia	6.5	Vietnam	0.1	Qatar	67.7	Ukraine	0.9	Philippine	11	Palestine	99
Pakistan	97	Turkmenistan	89	Jordan	92	Azerbaijan	96.9	Myanmar	4.15	Poland	0.1
Bangladesh	90	Kyrgyzstan	87	Lebanon	54	Armenia	0.1	Cambodia	99	Rumania	0.3
Sri Lanka	9.7	Tajikistan	98	Bahrain	70.2	Belarus	0.5	Laos	0.01	Czech	0.2
Afghan	99.7	Saudi Arabia	98	Yemen	99	Georgia	11	Brunei	79	Republic	5.7
Nepal	4.4	UAE	80	Albania	82.1	Moldova	1	East Timor	0.2	Slovakia	0.1
Maldives	100	Oman	25	Serbia	3.1	Montenegro	20	Kazakhstan	70.2	Estonia	0.1
Bhutan	0.1	Egypt	94.9	Macedonia	34	Ethiopia	33.9	Uzbekistan	90	Croatia	1.47
Mongolia	5	Kuwait	70	Bosnia	51	Kenya	11.1	Macedonia	34	Moldova	2
Indonesia	87.2	Thailand	4.9	New Zeeland	24	Panama	0.7	Korea	0.3	South Africa	1.9
Morocco	99.9	Hungary	0.05	Latvia	0.02	Lithuania	0.1	Slovenia	2.4	Bulgaria	8

Logistics is crucial for ensuring the quality of products throughout the supply chain, particularly in transportation, handling, and storage practices, until they reach the final consumer. The concept of halal assurance extends beyond merely distinguishing permissible from forbidden products; it also emphasizes the importance of high-quality compositions throughout the production process. Quality food encompasses both the religious imperatives of compliance and the principles of food safety, as Islam requires Muslims to consume only high-quality products while avoiding haram options (74, 84). Consequently, multiple aspects of the food supply chain, including farming, food manufacturing, and retail logistics, are significantly impacted by influences from non-Muslim nations and enterprises (71).

## 5 Conclusion and practical implications

The study evaluates the impact of video source links on the purchasing behavior of Muslim consumers within the OBOR supply chain network. It establishes that these video links significantly enhance demand sustainability for certified food products among Muslim consumers, particularly in several countries integrated into the OBOR initiative. The availability of traceable certified products fosters consumer confidence, reinforcing perceptions of higher standards and quality. Consequently, the study highlights that Muslim consumers are more inclined to purchase products that align with their religious beliefs, supported by visible evidence such as labels and video content. This trust, cultivated through verified product information, ultimately boosts consumer satisfaction and demand for certified products, thereby enhancing the sustainability of the market for high-quality nutritional offerings. The findings underscore the market opportunities for Chinese food firms across diverse Muslim regions linked to the OBOR initiative.

The traceability of certified products has significantly improved due to increased scrutiny within the food supply chain, particularly in relation to the OBOR initiative. Stringent handling practices throughout the entire supply chain ensure the provision of high-quality, nutritious, and safe products to Muslim consumers, meeting their specific requirements. This improvement boosts both product integrity and consumer confidence. The growing demand for certified products across various regions is further driven by the thorough investigations conducted by producers, wholesalers, and retailers. These internal assessments reinforce the quality assurance that certification inherently provides. By rigorously evaluating their own supply chains and products, these stakeholders not only mitigate risks associated with non-compliant goods but also clearly demonstrate their commitment to the standards represented by the certification. This proactive approach builds consumer confidence and, in turn, strengthens the appeal of certified products in the marketplace, creating a virtuous cycle of demand and quality assurance. Driven by increasing consumer awareness and the desire for safer, more sustainable food sources, Chinese food companies are actively adapting their practices. A key response has been a focused effort to increase the availability of certified products, indicating compliance with recognized standards of quality and origin. This initiative aims to foster consumer trust and address concerns regarding food safety, a common issue in the domestic market.

Furthermore, Chinese food companies are leveraging product videos to enhance traceability and promote market sustainability. These videos often showcase the journey of the product from farm to table, highlighting ethical sourcing practices and rigorous quality control measures. By providing visual evidence of responsible production, companies aim to foster transparency and connect with consumers who prioritize environmentally conscious choices. In conclusion, the adoption of certified product offerings and the utilization of product videos demonstrate a proactive approach by Chinese food companies to meet evolving consumer demands. By prioritizing traceability and promoting market sustainability, these businesses strategically position themselves to thrive in a market increasingly driven by ethical considerations and informed purchasing decisions. This study examines how Muslim consumers’ purchasing behaviors and perceptions of food are closely linked to their satisfaction with religious beliefs. Various factors inform Muslim consumers about the certification status of the products they choose, with particular emphasis on the distinction between legitimate, high-quality certification and issues of physical contamination.

### 5.1 Future research and limitations of the study

The growing significance of the Muslim market necessitates a more nuanced understanding of effective penetration strategies for firms seeking to engage this expansive demographic. While quality products remain paramount, fostering trust throughout global supply chains is equally crucial. Current sustainability practices, particularly those within the OBOR initiative, may not fully represent the optimal outcomes achievable through culturally sensitive and ethically grounded approaches. Therefore, further research is vital to explore how firms can deviate from existing models by focusing on enhanced trust and culturally relevant engagement, ultimately contributing to more sustainable and effective market penetration strategies across diverse global regions. Such research will not only deepen our understanding of market dynamics but also promote the development of more sustainable and responsible business practices in culturally diverse contexts.

The imperative for future research lies in rigorously assessing the safety and quality assurance of certified products across diverse regional marketplaces. This investigation is not merely academic; it is fundamental to upholding consumer trust and ensuring adherence to locally mandated standards. The variance in regional regulations and consumer expectations necessitates a deeper understanding of how certifications translate into tangible product safety and quality within specific contexts. Furthermore, enhancing traceability and sustainability within product supply chains offers another crucial avenue for exploration. By focusing on specific market regions, researchers can identify opportunities for optimization and develop tailored strategies that strengthen the integrity and reliability of product sourcing and distribution, ultimately contributing to a more responsible and transparent global marketplace. The study acknowledges certain limitations, particularly regarding the accessibility of technology among Muslim consumers. It is important to note that not all individuals within this demographic possess Android mobile devices or have reliable Internet access. Consequently, the ability to view product video source links may not exert a uniform influence on all consumers, potentially skewing the findings and implications of this research.

## Data Availability

The datasets presented in this study can be found in online repositories. The names of the repository/repositories and accession number(s) can be found in the article/supplementary material.
